# Deep infection following reconstruction of pelvic fractures: prevalence, characteristics, and predisposing risk factors

**DOI:** 10.1007/s00068-021-01618-y

**Published:** 2021-03-08

**Authors:** Nikolaos Konstantinou Kanakaris, Vincenzo Ciriello, Petros Zoi Stavrou, Robert Michael West, Peter Vasiliou Giannoudis

**Affiliations:** 1 Academic Department of Trauma and Orthopaedic Surgery, Leeds General Infirmary, Clarendon Wing, Floor D, Great George Street, Leeds, LS1 3EX UK; 2 Academic Department of Trauma and Orthopaedics, School of Medicine, Leeds Teaching Hospitals, University of Leeds, Leeds, UK; 3 Leeds Institute of Health Sciences, University of Leeds, Leeds, UK; 4 Trauma and Orthopaedic Surgery, School of Medicine, University of Leeds, Leeds, UK; 5 NIHR Leeds Biomedical Research Unit, Chapel Allerton Hospital, Leeds, UK

**Keywords:** Pelvic fractures, Surgical site infection, Risk factors, Cohort analysis

## Abstract

**Purpose:**

To identify the incidence, risk factors, and treatment course of patients who developed deep infection following fixation of pelvic fractures.

**Methods:**

Over a period of 8 years patients who underwent pelvic reconstruction in our institution and developed postoperative infection were included. Exclusion criteria were pathological fractures and infections that were not secondary to post-traumatic reconstruction. The mean time of follow-up was 43.6 months (33–144). For comparison purposes, we randomly selected patients that underwent pelvic fracture fixation from our database (control group). A logistic regression was fitted to patient characteristics including age, sex, ISS, and diabetic status.

**Results:**

Out of 858 patients, 18 (2.1%) (12 males), with a mean age of 41 (18–73) met the inclusion criteria. The control group consisted of 82 patients with a mean age of 41 years (18–72). The mean ISS was 27.7 and 17.6 in the infection and control group, respectively. The mean time from pelvic reconstruction to the diagnosis of infection was 20 days (7–80). The median number of trips to theatre was 3 (1–16). Methicillin-resistant *Staphylococcus aureus* (MRSA) was the most frequently isolated organism in the years prior to 2012. Eradication was achieved in 93% of the patients. The most important risk factors for deep infection were ISS (OR 1.08, 1.03–1.13), posterior sacral approach (OR 17.03, 1.49–194.40), and diabetes (OR 36.85, 3.54–383.70).

**Conclusion:**

In this retrospective case–control study, deep infection following pelvic trauma was rare. A number of patient-, injury- and surgery-related factors have shown strong correlation with this serious complication.

## Introduction

Pelvic ring fractures are potentially life-threatening injuries with high incidence of concomitant morbidity and mortality ranging from 5 to 60% in the literature [1–5]. The prevalence of pelvic fractures has been reported to vary from 20 to 37 cases/100,000 of the general population, affecting up to 20% of those with multiple injuries [1, 2]. Displaced and/or unstable fractures require surgical management, either in one or more stages following the principles of damage control orthopaedics (DCO) [6–8].

Temporary pelvic stabilisation is usually offered immediately after trauma in patients with suspected pelvic fractures in the form of pelvic binders [9]. Following initial resuscitation and diagnostics, patients with persisting hemodynamic instability and mechanically unstable fractures are treated as surgical emergencies with either external/internal fixation, pelvic packing, and/or embolisation [10–12]. Closed reduction techniques and mini-invasive pelvic fixation is offered to a large number of pelvic fractures by specialist trained orthopaedic trauma surgeons within the first few days [7, 13].

Open reduction and internal fixation are used in specific fracture types, as well as when closed reduction of the pelvic ring is ineffective, often due to prolonged delays of the definitive fixation [7, 14]. Definitive stabilisation often involves extensive surgical approaches and lengthy operative procedures [7, 15]. The immunosuppressive state induced by trauma [8, 16, 17], the presence of associated injuries, massive blood loss, the ICU stay, and the lengthy surgical interventions predispose these patients to both local and systemic infections [8, 18, 19].

Surgical site infection (SSI) is an infrequent complication following orthopaedic surgery, with a prevalence varying between 1 and 3% [20–22]. Risk factors for orthopaedic SSI can be grouped to patient-related (diabetes, obesity, smoking, older age, steroid-use or immunodeficiency), and to surgery-related (extended preoperative hospitalisation, massive intraoperative blood loss, prolonged operative time) [21]. Despite the low prevalence of SSI in trauma patients its consequences may result in complex and long lasting clinical problems including: prolonged hospital length of stay, doubled readmission rates, increased health-care costs (reaching +300%), and decreased overall physical and social functioning [22].

Trauma-related risk factors may also contribute to SSI developing in patients with pelvic injuries. Pelvic arterial embolisation, retroperitoneal packing, closed internal degloving (Morel-Lavallee lesion), urogenital and bowel tract injuries, and open pelvic fractures are all trauma-related factors that can contribute to the increase risk of local infection [12, 22–25].

The literature related to the development of SSI development following stabilisation of pelvic ring injuries is limited. Therefore, the aim of this study was to investigate the prevalence of surgical site infection following pelvic ring injuries in one of the largest tertiary referral centres in the UK; to delineate the pathogens and clinical characteristics of infections; the type and duration of treatment; the frequency of risk factors and general outcomes.

## Patients and methods

All consecutive patients who underwent surgical management (closed reduction mini-invasive fixation, or, open reduction and internal fixation) for pelvic injuries and developed surgical site infection, superficial or deep, over a period of 8 years (January 2006 to December 2014), were eligible for inclusion in the study. Patients with pathological fractures or with pelvic osteomyelitis not related to the surgical procedure (haematological diffusion, endopelvic infection), patients with pure acetabular fractures, as well as, those with superficial external-fixator pin site infections were excluded from this study.

The adopted definition of SSI (superficial incisional, deep incisional or organ/space SSI) was in accordance with the Centres for Disease Control and Prevention/National Nosocomial Infections Surveillance system [26, 27]. More specifically, an SSI was characterised as “superficial incisional” when it occurred within 1 year after the operative procedure and involved only the skin and subcutaneous tissue of the incision. In addition, at least one of the following was present: purulent drainage from the superficial incision; organism isolated from an aseptically obtained culture of fluid or tissue from the superficial incision; and at least one of the following signs or symptoms of infection—pain or tenderness, localized swelling, redness or heat, and the superficial incision was deliberately opened by the surgeon [26].

The SSI was characterised as “deep incisional” when the following criteria were fulfilled: the infection occurred within 1 year after the operative procedure and the infection involved deep soft tissue (e.g., fascial and muscle layers) of the incision. In addition, at least one of the following criteria: purulent drainage from the deep incision but not from the organ/space component of the surgical site; a deep incision that spontaneously dehisced or was deliberately opened by the surgeon, and the patient had at least one of the following signs or symptoms—fever (> 38 °C), localized pain, or tenderness, or evidence of an abscess or of infection involving the deep incision found on direct examination, during reoperation, or by histopathology or radiologic examination [26].

The SSI was defined as “Organ/Space SSI” when the following criteria were met: the infection occurred within one year after the operative procedure and the infection appeared to be related to the operative procedure and involved any anatomical site other than the incision, or areas opened or manipulated during the operative procedure.

In addition, at least one of the following criteria had to be met: purulent drainage from a drain into the organ/space; organisms were isolated from an aseptically obtained culture of fluid or tissue in the organ/space; an abscess or other evidence of infection involving the organ/space was found on direct examination, during reoperation, or by histopathology or radiologic examination [26]. An infection that involved both superficial and deep incision sites was classified as deep incisional SSI. When there was organ/space infection that was draining through the incision and did not involve reoperation it was considered as a deep incisional SSI [26].

A prospectively compiled computerised database (from the theatres’ information system and the hospital admission tracking system) including all the patients with a pelvic ring fracture was retrospectively reviewed for clinical and radiological data including patient characteristics, injury severity, surgical interventions, complications and infection characteristics. Patient’s characteristics included: demographic data, the presence of significant co-morbidities, and risk factors including diabetes mellitus, obesity, malnutrition, smoking, drug use, alcoholism (> 14 units per week) [28], vasculopathy, tumours, steroid-use, and known immunodeficiency. Injury characteristics included: mechanism of injury (MoI); type of pelvic injury and the presence of associated acetabular fracture; presence of associated extra-pelvic injuries; open or closed injury pattern; the presence of Morel-Lavallee lesions (MLL); the presence of urogenital lesions; and number of blood units transfused. Surgery characteristics included: type of surgery and time elapsed between injury and procedure; pelvic arterial embolisation; pelvic packing; duration of surgical procedure; and peri-operative blood loss, complications, and mortality. Infection characteristics included: site and type of infection (superficial/deep; acute/chronic); microbiological aetiology; modality and time of diagnosis; type, modality and duration of antibiotic treatment; modality and number of surgical wash-out and debridement; need of implant removal; usage of vacuum assisted closure (VAC); and need of plastic surgery and eradication of infection. The microbial cause of SSI was considered to be caused by the pathogen(s) identified at the time of first surgical debridement/exploration of the pelvis post diagnosis of the infection. Other data documented included total length of in-hospital stay (LOS), ICU length of stay and time to bony union.

All pelvic SSIs were discussed at the multidisciplinary meeting for pelvic trauma of the department including specialist microbiologic input [29]. Initial management included prompt surgical debridement and copious irrigation with normal saline solution (at low pressure and without antimicrobial agents) of the infected site. Empirical antibiotic therapy was initiated after obtaining tissue samples at time of debridement. The empirical antimicrobial regimen was either continued or modified according to the culture results and local microbiology guidance. Debridement was repeated as necessary based on the clinical and biochemical parameters. The frequency and number of debridement was directed by the severity and the extent of the infection, degree of local and surrounding soft-tissue damage, and the virulence of the implicated micro-organism. The duration and the route of administration of the antibiotics were dependent on the patient’s systemic and local response. Metalwork was removed when mechanical stability was restoredor when it was exposed without an option of coverage. In case of severe soft-tissue defects the use of Vacuum Assisted Closure (VAC therapy), or plastic surgical reconstruction were adopted [30].

Fractures were classified according to Young and Burgess System (Anterior Posterior Compression (APC), Lateral Compression (LC), Vertical Shear (VS) and Combined Mechanism of injury (CMI) [31]. This study was approved by the institutional review board (Number TRS/15/012). The mean follow-up period was 43.6 months (33–144).

A control group of operatively treated pelvic fractures that did not develop an SSI was randomly identified from the same database to explore the impact of 29 risk factors to the development of such a complication (Table 1). The control group consisted of patients treated at the same unit over the same period of years, having a complete dataset available and their surgery within ± 7 days from a case of the control group.

**Table 1:** The basic characteristics of the two groups are presented

Characteristics	Patients with SSI *n*, %	Patients without SSI *n*, %	*p* value
Number of patients	18, 100%	82, 100%	
Gender ratio male/female^a^	12/6	52/30	1.000
Age mean (SD)^a^	41 (20.5)	40.0 (17.9)	0.793
ISS mean (SD)^a^	28 (13)	18 (10)	**< 0.001**
Pelvic fracture types—Young Burgess LC/APC/VS/CMI^a^	9/2/4/1	47/21/1/0	**0.001**
Combined cases with acetabulum: acetabular fracture types—letournel simple/associated types^a^	1/4	3/13	1.000
Open fracture^a^	4, 22.2%	4, 4.9%	**0.048**
Morel-Lavallee^a^	3, 16.7%	6, 7.3%	0.423
Diabetes^a^	4, 22.2%	1, 1.2%	**0.002**
Obese^a^	8, 44.4%	8, 9.8%	**0.001**
Smoker^a^	4, 22.2%	14, 17.1%	0.860
High alcohol intake (> 14 units per week)^a^	5, 27.8%	6, 7.3%	**0.036**
On steroids^a^	1, 5.6%	2, 2.4%	1.000
Immuno-compromised^a^	2, 11.1%	2, 2.4%	0.300
Duration of surgery, mean (SD)^a^	136 (50.3)	110 (44.5)	**0.028**
External fixation^a^	10, 55%	30, 36.6%	0.430
Ilio-sacral screws^a^	15, 83.3%	61, 74.4%	0.617
Plates^a^	9, 50%	47, 57.3%	0.761
Open reduction^a^	10, 55.6%	47, 57.3%	1.000
Mini invasive percutaneous techniques^a^	15, 83.3%	60, 73.2%	0.548
Ilioinguinal^a^	2, 11.1%	10, 12.2%	1.000
Pfannenstiel^a^	5, 27.8%	25, 30.5%	1.000
Kocher–Langenbeck^a^	1, 5.6%	8, 9.8%	0.913
First window IL^a^	1, 5.6%	8, 9.8%	0.913
Posterior sacral^a^	3, 16.7%	1, 1.2%	**0.018**
Urogenital injury^a^	4, 22.2%	8, 9.8%	0.283
Embolisation^a^	3, 16.7%	3, 3.7%	0.120
Pelvic Packing^a^	1, 5.6%	0, 0%	0.403
Units transfused, mean (SD)^a^	4.3 (4.2)	2.9 (4.2)	0.194
Other surgeries^a^	11, 61.1%	21, 25.6%	**0.008**
LOS in days, mean (SD)	46.3 (22.4)	17.8 (15.7)	**< 0.001**
ICU LOS in days, mean (SD)	9.6 (5.0)	4.2 (4.7)	**< 0.001**
Pelvic nonunions	2, 11.1%	1, 1.2%	0.143
Urogenital complications	5, 27.8%	6, 7.3%	**0.036**
LRTI	8, 44.4%	6, 7.3%	**< 0.001**
ARDS	5, 27.8%	0, 0%	**< 0.001**
VTE events	2, 11.1%	9, 11%	1.000
Mortality	1, 5.6%	2, 2.4%	1.000
Independent mobilisation at last follow up	10, 55.6%	69, 84.1%	**0.017**
Return to preinjury mobility state	12, 66.75	70, 85.4%	0.126
FUP in months, mean (SD)	32.0 (19.8)	24.7 (8.8)	**0.016**

^a^Refers to the 29 risk factors for pelvic surgical site infection, which have been investigated via the different models of logistic regression analysis as described at the “Patients and Methods”

### Statistical analysis

Initially, a base model, a logistic regression, was fitted to patient characteristics including age, sex, injury severity score, and diabetic status. For continuous variables such as age and ISS, nonlinearities were investigated. It was required that there was good evidence (at the 5% significance level) for all factors in the base model and the base model was checked using fivefold cross-validation. Secondly, the base model was supplemented by each surgical risk factor in turn. Statistically significant surgical factors were then added to the final model. Again nonlinearities were explored for continuous risk factors such as number of transfused units of blood and fivefold cross-validation was employed as a further check. Odds ratios for the statistically significant factors in the final model were reported along with 95% confidence intervals. A Receiver–Operator Characteristic curve was determined for the final model and sensitivity and specificity reported.

## Results

During the study period 858 patients were operated in our regional major trauma centre with the diagnosis of pelvic ring injuries excluding pure acetabular fractures. Out of those, 18 (2.1%) patients developed SSI (study group). The gender ratio was 2:1 (male/female), the mean age was 41.2 years (17–73). Eight patients (44%) were obese (BMI > 30); one with malnutrition (6%); four patients (22%) were smokers; and two patients were intravenous drug-users. Four patients (22%) had history of diabetes.

The most common mechanism of injury was road traffic accident (14, 78% of cases), followed by fall from height (3, 17% of cases) and simple falls (1, 6%). According to Young and Burgess system [31], LC injuries were identified in 8, 44%, APC in 6, 33.3% and VS in 4, 22% of cases. Four patients (22%) presented with a combined pelvic-acetabular fracture. Four patients (22%) presented with an open pelvic injury having deep perineal lacerations. In two cases, the laceration involved the anal sphincter and required surgical repair. Four patients sustained urogenital injuries. Morel-Lavallee injuries were present in three (16.7%) patients (lateral and posterior aspect of thigh in two patients; sacral and groin region in one patient). All the lesions were debrided at the time of pelvic fixation. Extensive perineal haematomas were present in four patients (22%).

Associated non-pelvic injuries were present in 12, 67% of patients, with an ISS > 16 in 50% of these cases. One patient had a head injury; maxillo-facial injuries were present in five patients; pneumothorax in four and abdominal trauma in two (one had liver and spleen lacerations; one had bowel rupture). Two patients sustained vertebral fractures without spinal cord compromise. Six patients (33%) and four (22%) had associated lower and upper limb injuries, respectively,

DCO was performed on arrival in 9 (50%) cases to stabilize the pelvic injury and control haemorrhage. In the other patients early total care (ETC) was performed within 24 h from their accident. Pelvic arterial embolisation was performed in three patients to control persisting arterial bleeding as identified at the initial trauma scan. One patient underwent retroperitoneal pelvic packing. Seven patients (39%) required secondary surgical treatment after the initial DCO.

The mean number of blood units transfused during the first 48 h from the time of admission was 4.3 units (0–16). The median time elapsed between injury and the definitive surgical procedure was 14 days (2–25 days). Four patients (22%) had primary delayed surgery at the median of 9 days after injury (2–42). The median operative time for definitive surgical procedures was 136 min (70–495 min). In all visits to theatres, all patients received antimicrobial prophylaxis according to the guidelines of the department (single dose of flucloxacillin 1 g and gentamicin 2 mg/kg iv). In high-risk cases for MRSA or patients allergic to penicillin, teicoplanin 400 mg iv was administered instead of the flucloxacillin.

The median time elapsed from pelvic reconstruction to SSI diagnosis was 20 days (7–80). In five patients (28%) SSI complicated pubic symphysis plating; in three patients (17%) SSI was localized to the SI screw site; in two patients (11%) SSI affected both pubic symphysis and sacro-iliac site and in one patient involved both iliac crests (pin sites) and the SI joint site.

Out of the four patients who sustained combined pelvic and acetabular fractures, one of them was treated with the insertion of SI screws and axial traction (trans-condylar distal femoral traction) due the extensive soft-tissue necrosis affecting the ipsilateral gluteal and the groin area as a consequence of the MLL and pelvic artery embolisation. This patient developed a widespread deep pelvic and acetabular infection, involving even the site of SI screw.

The most frequent aetiological agent causing SSI in our series was methicillin resistant *Staphylococcus aureus* (MRSA) isolated in 10, 56% of patients (all in patients treated between 2005 and 2012). Mono-microbial infections (11 cases, 61%) of which 7 cases were attributed to MRSA, and in a single case each *Escherichia coli* (*E. coli*), coagulase negative *Staphylococcus* (CoN Staph), *Pseudomonas aeruginosa* and methicillin sensitive *Staphylococcus aureus* (MSSA) were isolated. Polymicrobial infections affected seven patients in the entire cohort (39%). Coliform bacteria were isolated in six of these cases in combination with anaerobic species in three patients, MRSA and *Pseudomonas aeruginosa* in another two, whilst one patient had MRSA and *E. coli* polymicrobial SSI.

All the patients received antibiotic therapy based on the culture sensitivity for a median period of 6 weeks (1–9 weeks) in accordance to the expert microbiology advise. The median number of trips to theatre for wash-out and surgical debridement was 3 (1–16). Wound closure was achieved with the help of vacuum assisted closure (VAC) in 10, 56% of patients. Bony union was achieved in all but one patient, 94%. Metalwork removal was necessary in 10 patients, 56%.

Eradication of infection was achieved in all patients except one (93%) that developed chronic osteomyelitis of his right hemi pelvis. This patient, as mentioned above, sustained an extensive necrosis of soft tissue and required a rectus abdominis transposition flap to cover the pelvic defect. After 16 trips to theatre, he refused further surgical debridement and decided to manage the infection only with oral antibiotic therapy as necessary.

SSI following pelvic ring injuries resulted to lengthy hospital stays with a median of 51 days (23–115 days) per patient. Six patients, 33%, required ICU support with a median ICU stay of 6 days (1–9 days). Two patients developed coexistent pneumonia, treated and resolved with oral antibiotic therapy. No further major complications were observed.

At the final follow-up, mean time of 43.6 months (33–144), 16 patients (89%) were fully mobile and ambulating without any means of assistance. Of the remaining two patients, one developed Brooker [32] type IV heterotrophic ossification requiring surgical excision (30 months after the index accident); the other one was the patient with unresolved infection. No deaths were noted in the cohort of pelvic SSIs.

For the analysis of the suspected as risk factors for an SSI, the randomly selected control group of operatively treated pelvic fractures without developing an SSI consisted of 82 patients. Their characteristics are presented at Table 1.

Exploration of the study and control cohort of patients with pelvic fractures using logistic regression has revealed that there are strong associations between site-specific infection and the following factors (Table 2):

- Injury Severity Score (ISS),
- Diabetes,
- Posterior sacral fixation,
- Alcohol intake.

**Table 2:** The final model of the logistic regression analysis resulted to the presented data

Risk factor	OR	95% CI
ISS per unit	1.08	(1.03, 1.14)
Diabetes	45.82	(4.02, 522.64)
Posterior sacral	18.86	(1.46, 243.38)
Alcohol	5.39	(1.06, 27.60)

## Discussion

The development of SSI following orthopaedic and trauma surgery is not common and varies between different anatomical sites and surgical approaches. The incidence of SSI varies from 0.7% in patients undergoing hip replacement to 7.9% in patients undergoing spinal fusion [18]. The SSI rate has been reported previously as 5.7% following pelvic ring fractures fixation and 5.2% after acetabular fracture fixation [22, 33]. In this study, the prevalence of SSI following pelvic ring injury was 2.1%.

Prevention of SSIs in trauma and orthopaedics has evolved significantly over the last decades [34–36], and include several pre-, intra-, and post-operative measures. Among these, screening for potential carriers, source isolation of positive patients, and decolonisation for MRSA of all acute admissions have been part of our hospital routine since 2011. The high prevalence of this difficult to treat bacteria in our cohort of pelvic SSIs, can be attributed to the absence of such measures at the earlier years, as only one SSI linked to MRSA was identified after 2012 in this series.

Multiple risk factors for orthopaedic surgical site infections have been identified [37–39]. Several medical co-morbidities including rheumatoid arthritis, diabetes, and urinary tract infection have been reported to increase the risk of an SSI following orthopaedic procedures [37, 40]. Despite the relatively young age of the high energy pelvic trauma patients, as in this series, diabetic patients were at significant risks for deep infection following their pelvic fixation (*p* = 0.002).

Other previously reported general factors, as the use of tobacco products, corticosteroids, methotrexate or non-steroidal anti-inflammatory medication therapies and immunodeficiency were not highlighted in this cohort of pelvic fracture patients [37, 40]. In addition, obesity and malnutrition have been both associated with higher rate of wound complications and SSI [41, 42]. In this series, the obesity was statistically significantly higher in the study group, however, following logistic regression failed to demonstrate strong correlation.

With regard to surgery-related risk factors for SSI, evidence deriving from elective orthopaedic procedures or spinal surgery, although relevant are less specific for trauma or fracture related procedures [36, 37, 43]. Extended preoperative hospitalisation, massive intraoperative blood loss, and prolonged operative time have been shown to be associated with an increased rates of SSIs. This was partially verified in the base analysis of our study in regards to the duration of surgery (*p* = 0.028), but was not documented following logistic regression.

The presence of open pelvic trauma and the severity of associated trauma reached both statistical significance at the base model as risk factors, (*p* = 0.048 and *p* < 0.001, respectively). Other factors as the history of pelvic embolisation, pelvic packing, use of external fixators/C-Clamps, the presence of a suprapubic catheter, or of Morel-Lavallee lesions or the large number of transfusions and evidence of post-traumatic immunoparesis were not confirmed in this study in contrast to the previous reports. [44–49]. These differences could be attributed to the small incidence of SSI in our cohort, leaving only 18 cases of recorded deep infection to analyse.

The Morel-Lavallee lesion, a closed degloving injury described in the mid-nineteenth century [23, 50], is a result of shear forces applied to the soft tissues that separates the skin and subcutaneous tissue from the underlying fascia, creating a cavity. The bleeding from the disrupted perforating arterial plexus collects in the cavity forming a hematoma. The presence of necrotic tissue and hematoma in the subcutaneous layers has been reported to increase the risk of infection [22, 23, 51]. Hak et al. in a case series of 24 patients reported positive cultures in 46% of cases at the time of the initial debridement. Suzuki et al. showed an eightfold increase in the relative risk of developing SSI following acetabular fracture fixation in the presence of MLL [21]. Tseng and Tornetta in a recent study [51] described a percutaneous technique to manage MLL and advocated the early debridement of these lesions. The authors suggested that percutaneous procedures for pelvic fixation can be well tolerated in the same operative setting of debridement. Open pelvic reconstructions should be delayed [51]. In our series, although we observed MLL lesions in nine overall cases, they were not associated with an infection, probably as part of our strategy avoiding surgical approaches through the degloved areas and the use of less invasive fixation techniques.

In our cohort, the patients who underwent pelvic arterial embolisation (PAE) showed the worst outcomes in terms of number of surgical procedures required to control SSI and hospitalisation. Arterial embolisation represents an efficient acute intervention to control severe arterial bleeding following pelvic trauma [10] but potential complications include: gluteal muscle necrosis and skin ulceration, although these are reported to be rare in the literature [10, 52]. Manson et al. recently reported an increase of deep infection rate after acetabular fracture fixation (up to 58% of cases) in patients who previously underwent pelvic angiographic embolisation. The authors suggested avoiding embolisation of the entire iliac artery whenever possible; especially in case of coexistent acetabular fracture that needs ORIF [24]. One patient in our cohort with an acetabular fracture underwent PAE. The fracture was managed conservatively because the patient developed gluteal necrosis and skin ulceration as a consequence of PAE. A rotational flap was necessary to cover the soft-tissue defect, but the patient subsequently developed chronic osteomyelitis of the left hemi-pelvis.

Preperitoneal pelvic packing is a rare damage control procedure reserved for patients in extremis with exsanguinating pelvic trauma. It can be effective under these circumstances, contributing to the successful resuscitation of the bleeding patient and subsequent leads to secondary definitive fixation of the pelvic ring. Historically, it has been advocated that this procedure is associated with surgical site infections and subsequent morbidity [12]. However, a recent retrospective controlled study found similar SSI rates (8%) after preperitoneal pelvic packing when compared to single stage anterior pelvic fixation (9.2%) [53]. In the present study, one patient that developed an SSI located at the retropubic space of Regius, who had undergone preperitoneal packing at the time of admission.

However, in our analysis, both PAE and Pelvic Packing were not identified as risk factors for deep infection following pelvic trauma surgery (*p* = 0.12 and *p* = 0.403, respectively), most likely due to their infrequent use and the small size of our overall sample.

Favourable outcomes were noted with eradication of the infection achieved in all but one patient. Early diagnosis, prompt and accurate surgical debridement and appropriate antibiotic therapy appeared to be the key of success in pelvic SSI management. The length of stay for SSI reported in our series is higher compared to those reported by Whitehouse et al. (median 51 vs. 14 days) [22]; however, the Whitehouse study included 59 patients of which only 8 sustained traumatic injuries. The majority of the patients included underwent elective surgical procedures. The more complex nature of injuries reported in our cohort could explain their prolonged hospitalisation compared to that reported by Whitehouse et al. [22].

The current study has some limitations. The small sample size and the retrospective nature of the study are the main ones. The findings reported here are from an exploration of an observation dataset based on a convenience sample. The sample size is limited with only 18 infections reported so that the analysis cannot be regarded as robust. In particular, potential risk factors which did not show statistical significance cannot be disregarded for future study. Since there are at least 29 potential risk factors for SSI and only 18 events, a robust investigation of all risk factors was not possible. A suitable approximate rule is that 10 events (infections) are needed for every risk factor that is to be studied, indicating that a cohort of around 300 SSI following pelvic and/or acetabular fracture reconstruction would be necessary to be analysed in comparison to at a least an equal non-infected cases as a control group.

Instead, an exploration of risk factors was undertaken using the study group of 18 infected cases and a fourfold cross-validation control group. This work has identified four risk factors, mainly, ISS, diabetes, posterior sacral fixation, and alcohol intake. Consequently, when these factors are present special care should be taken to minimise the risk of infection. Strengths of the study include the expertise of a specialist pelvic and acetabular unit by the same two surgeons, and the prolonged continuity of care of the patients and long-term follow-up of this cohort.

In conclusion, the prevalence of the SSI for pelvic ring injury stabilisation is low. Risk factors verified to be associated with deep infections included the high overall severity score, the presence of diabetes, alcohol consumption, as well as the need for posterior approaches for pelvic ring fixation. Even though the final outcomes appeared to be satisfactory, SSI development resulted in increased LOS and hospitalisation rate. Patients with SSI underwent multiple surgical procedures and prolonged antibiotic therapy to control and to eradicate the infection. Early diagnosis and appropriate management of SSI need a multidisciplinary team approach. Further studies with higher sample size are desirable to identify additional risk factors and to report on the outcomes of these patients.
